# Forecasting malaria incidence based on monthly case reports and environmental factors in Karuzi, Burundi, 1997–2003

**DOI:** 10.1186/1475-2875-6-129

**Published:** 2007-09-24

**Authors:** Alberto Gomez-Elipe, Angel Otero, Michel van Herp, Armando Aguirre-Jaime

**Affiliations:** 1 Public Health Department, Universidad Autónoma de Madrid, C/Arzobispo Morcillo 2, 28029 Madrid, Spain; 2 Department of Epidemiology, Médecins Sans Frontiéres, 94 rue Dupré, 1090 Brussels, Belgium; 3 Research Support Service, NS Candelaria University Hospital, Ctra. Gral. del Rosario s/n, 38010 Santa Cruz de Tenerife, Spain

## Abstract

**Background:**

The objective of this work was to develop a model to predict malaria incidence in an area of unstable transmission by studying the association between environmental variables and disease dynamics.

**Methods:**

The study was carried out in Karuzi, a province in the Burundi highlands, using time series of monthly notifications of malaria cases from local health facilities, data from rain and temperature records, and the normalized difference vegetation index (NDVI). Using autoregressive integrated moving average (ARIMA) methodology, a model showing the relation between monthly notifications of malaria cases and the environmental variables was developed.

**Results:**

The best forecasting model (R^2^_adj _= 82%, p < 0.0001 and 93% forecasting accuracy in the range ± 4 cases per 100 inhabitants) included the NDVI, mean maximum temperature, rainfall and number of malaria cases in the preceding month.

**Conclusion:**

This model is a simple and useful tool for producing reasonably reliable forecasts of the malaria incidence rate in the study area.

## Background

Each year malaria affects over 100 million persons worldwide, with an annual cost in human life exceeding one million deaths, mainly children under five years of age in sub-Saharan Africa [1]. The number of disability-adjusted life years due to malaria, a measure of disease burden, was estimated at 46,486,000 for 2002, 87.8% of which was in sub-Saharan Africa [2]. Because of its strong epidemic potential, malaria continues to be an important public health problem in communities in semi-arid areas and in the highlands of Africa. These populations are exposed to factors that strongly influence the origin and magnitude of malaria epidemics, such as weakened immunity of the population associated with famine and massive displacements, failures of control measures and epidemiologic disease surveillance, and unstable environmental factors such as rainfall, temperature and vegetation [3]. There exist settings where malaria behaves as endemic malaria and other ones where it does as epidemic malaria. The attack rate and the case fatality rate of the malaria epidemics are also related with the level of partial immunity to malaria due to the previous exposition of the population to this infection that in the case of epidemic-prone settings, as the African highlands, is very low or even null. The collapse of health services during epidemics has been estimated to increase case fatality rate for severe malaria up to 25–50% [4]. It has been estimated that the population living in malaria epidemic-prone areas in Africa, nearly 125 million persons, suffers some 12.4 million malaria episodes per year attributable to epidemics, or about 4% of the annual number of malaria cases occurring worldwide [5].

Due to the severe health impact of malaria epidemics there is a growing need for methods that will allow forecasting, early warning and timely case detection in areas of unstable transmission, such as the African highlands, so that more effective control measures can be implemented [5-7]. Studies of malaria epidemics in these areas have shown their association with excess rainfall, temperature and vegetation density measured by the normalized difference vegetation index (NDVI). This is seen in the direct correlation between an abundance of *Anopheles *mosquitoes and rainfall [8,9], increased transmission and temperature [10], and vegetation density and malaria seasonality [11,12].

In late 2000, an epidemic of malaria from *Plasmodium falciparum *occurred in Burundi, with reported attack rates exceeding 200% and an estimated annual malaria-specific mortality of 1.6% (95% CI 0.9 – 2.8%) in children under five years of age [13]. The present study focuses on the province of Karuzi, in the highlands of Burundi. The hypothesis stated in this paper is that malaria incidence in a particular month can be predicted by rainfall, temperature and vegetation density – as factors that determine the density and infectivity of *Anopheles *mosquitoes – and the malaria incidence in the preceding month – as an estimator of the human reservoir of the parasite and of population susceptibility. The objective of this work was to study the relation among these variables to develop a predictive model that can forecast the incidence of malaria with reasonable reliability using the reported case rate, rainfall, temperature and vegetation density.

## Methods

### Study Area

Karuzi is a province in the central-eastern part of Burundi, at an altitude of 1500 – 1900 m. The province has a tropical climate characterized by a rainy season from October through April and a dry season from May through September, with mean annual minimum temperatures of 10.5 – 13°C and maximum temperatures of 25.5 – 28.5°C. Vegetation in the province consists primarily of palm and banana trees, with pine forests in the hills and cereal crops in the valleys. The mean NDVI is 0.36 from July through October and 0.53 from November through June. The population of Karuzi is 302,062, and the province is subdivided into seven communes. The health infrastructure consists of one 100-bed hospital and 11 health centers with a total of 311 beds [14].

### Data collection

#### Incidence of malaria

A case of malaria was defined as a patient seeking medical attention with fever over 38°C and no signs of acute respiratory infection, urinary infection, otitis, meningitis, measles or abscesses. This is the definition for a "case of malaria" used in all Burundi health facilities at the time of the study and for notification purposes. No changes of this definition were performed during the study period. Only 5 – 20% of clinical cases had microbiological confirmation in non-epidemic periods and no more than 2% during outbreaks, depending on the health facility. The health services compile all notifications of malaria consultations on a monthly basis. The cumulative number of notifications is as the numerator for the incidence rate, and the denominator is the total population of the province according to the population census adjusted for the growth factor which is 1.32 for the period 1995–2000 and 3.29 for the period 2000–2005 [15]. For this study there were used the 1997–2003 series of these incidence rates per 100 inhabitants of Karuzi.

#### Rainfall

Monthly cumulative precipitation for 1997–2003 was obtained from the Karuzi meteorological station of the Burundi Geographic Institute, measured in millimeters of rain fallen.

#### Temperature

Minimum and maximum monthly temperatures for 1997–2003, measured in degrees centigrade, were obtained from the same source as the rainfall data.

#### Vegetation density

The mean NDVI per month in Karuzi for 1997–2003 was obtained from images taken with the Advanced Very High Resolution Radiometer (AVHRR) sensor on board the National Oceanographic and Atmospheric Administration satellites, with a resolution of 8 km, on a scale of 0–0.7 [16].

### Epidemiological assumptions

The relation between malaria transmission and various factors was described by MacDonald in 1957 [17]. The main factors affecting transmission are vector population density, transmission capacity (based on vector survival and duration of the extrinsic incubation period – EIP) and immunity of the susceptible human host. Other factors such as strain virulence are of negligible importance. Of the meteorological data available in this study, rainfall influences the vector population (by increasing the capacity of larva production and maturation) and is reflected in the vegetation index, and temperature influences the transmission capacity (with higher temperatures shortening the EIP). This hypothesis is based on the fact that in tropical areas at altitudes over 1200 m, the most important factor limiting malaria transmission is the minimum temperature, because parasite development (sexual reproduction and development of sporozoites) is interrupted at temperatures lower than 16°C.

Generally, there are tropical areas between 25N-25S latitudes, at elevations of 1000–2000 m that have enough rainfall to maintain abundant marshy areas where the vector larvae develop, so that rainfall is often not the limiting factor. In epidemic situations, some of the factors that could plausibly explain fluctuations in transmission are: 1) higher minimum temperature, permitting prolongation of seasonal transmission and a "staircase" effect of repeated superinfections with increased parasitemia and anaemia up to clinical thresholds; 2) higher maximum temperature, shortening the EIP and producing an exponential effect on vector transmission capacity; 3) more abundant rainfall, with a consequent increase in vegetation density, resulting in a larger vector population and a linear increase in transmission, and 4) increased population reservoir of the parasite, which induces increased speed of transmission.

### Modelling assumptions

Taking the above-mentioned epidemiological assumptions into account, the following general form of the model is proposed to estimate the expected malaria incidence rate: let *I*_*t *_represent the malaria incidence rate in month *t*; *R*_*t *_is the cumulative level of precipitation for that month; *T*_*t *_is either the mean minimum temperature or mean maximum temperature for that month; *V*_*t *_is the mean vegetation density for that month, *p *is the seasonal period of oscillation for the previous three variables; and, *I*_*t*+*k *_is the malaria incidence rate for a future month that is *k *months from *t*. Then, the relation of influence among these variables, remain as follows:

∑*αI*_*t *_>* ∑*β*sin [(2*π/p*)*R*_t _* * T*_t _* *V*_*t*_] → *I*_*t+k*_

This relation expresses that a linear or cumulative combination of previous values of the incidence rate, as an estimator of population reservoir, and the cumulative combination of past levels of rain, temperature and vegetation density, as estimators of vector capacity, combine to influence future values of the incidence rate. The term that includes rainfall, temperature and vegetation density implies that the malaria incidence oscillates with a period that is proportional to their common seasonality. In expression (1) α is the linear regression coefficient for the incidence rate, and β is a parameter that determines the amplitude of seasonal oscillation estimated by regression. The use of * as an operator to link the components expresses the lack of *a priori *knowledge of how they are interrelated – interrelations that will be determined by trial and error. The model combines all those terms having significant autocorrelation and cross-correlation coefficients with the incidence rate in their corresponding lags at a significance level of p ≤ 0.05.

### Data processing

The following steps were carried out: a) exploration of serial incidence rates, temperatures, precipitation and vegetation, to identify regularities; b) trend analysis and periodogram of the incidence rate with Fast Fourier and Tukey Transforms to identify the periodic oscillations to be modeled, so the last seasonal periods sub-series gets separated for validation purposes, shortening also the rainfall, temperature and vegetation series accordingly; c) correlograms of the simple autocorrelation function (ACF) and partial autocorrelation function (PACF) for the incidence rate, with lags equal to their period of oscillation. Identification, adjustment and evaluation of the autoregressive integrated moving average (ARIMA) equation that explains the rate by its previous values, to use as a term in model (1); d) periodograms with Fast Fourier Transform of serial rainfall, temperature and vegetation, to identify seasonal oscillations and their period *p *in (1), and cross-correlations of these three data series with the ARIMA residuals in the serial incidence rate to identify lags in the influence; e) combination of ARIMA terms and oscillatory component to shape model (1), and estimation of the linear regression coefficients of the terms and goodness-of-fit of the model; f) successive entry in the model of serial incidence rates, rainfall values, temperature and vegetation, in their corresponding lags, to obtain the expected malaria incidence rates for each point of the temporal window of the series; g) the model was tested using the sub-series of malaria incidence rates separated, and its reliability was tested by comparing each predicted rate with that observed for the corresponding month.

The reliability criteria for the forecast consisted in verifying that: i) the difference between the predicted and observed value is white noise, or a normal random variable with a mean of 0 and standard deviation of 1; the randomness of the difference is tested based on the periodogram of the data and the runs test, and normality is tested with the histogram and the Kolmogorov-Smirnov test; ii) the differences do not exceed the limits of the 95% confidence interval by more than 5%; values falling outside the confidence interval are counted in the scattergram of the difference (y axis) with respect to the observed rate (x axis); and iii) the differences do not tend to increase or decrease when the observed rate increases, that is, the precision of the forecast does not depend on the magnitude of the rate. To test this, the correlation between the difference and the observed rate is estimated by the Pearson linear correlation coefficient. Trend analysis is used to test the statistical significance of the slope of the trend of the difference with respect to the observed rate.

The images of the vegetation index were processed using WinDisp 4. Data processing was performed using the statistical packages SPSS™ 13.0 from SPSS Co., and Statgraphics Plus^© ^5.1 from Statistical Graphics Co. A 2-tailed significance level of 0.05 was established for all tests.

## Results

**Figure 1:**
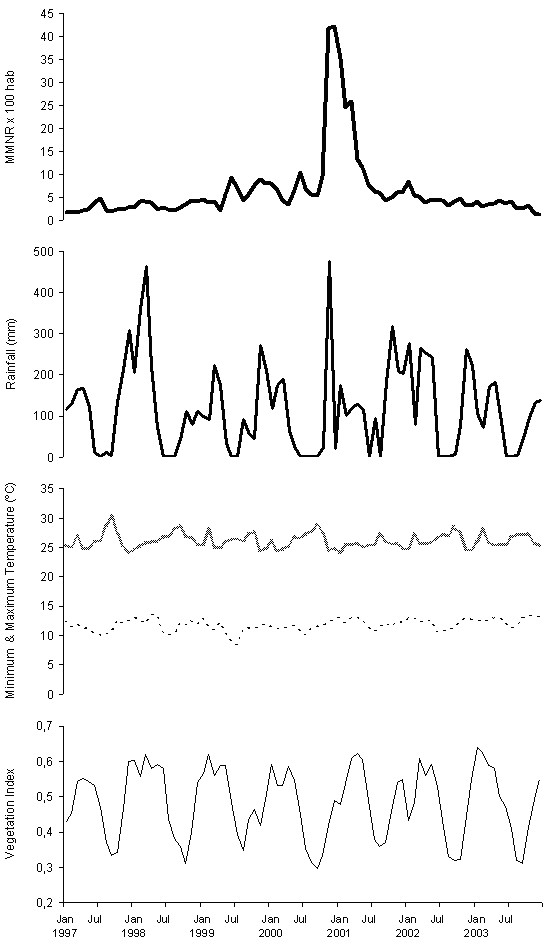
Monthly malaria notification rates (MMNR) per 100 inhabitants in Karuzi province, Burundi, from January 1997 to December 2003 (heavy bold solid line); monthly cumulative rainfall in mm (light bold solid line); maximum monthly temperature in °C (bold dashed line); minimum monthly temperature in °C (light dashed line); and normalized difference vegetation index-NDVI (light solid line).

Exploration of the malaria rate, precipitation, temperatures and vegetation for 1997–2003 shows no clear trend, and suggests a seasonal dependency in the series, with a 6-month period for the rate, and a 12-month period for rainfall, temperatures and vegetation, indicating a rhythmic oscillation of the four variables (Figure 1).

According to the results of the trend analysis and periodograms, the rate shows no trend or periodic oscillation. The 1997–2001 period is taken as base series for the incidence rate, and the same for rainfall, temperature and vegetation, leaving as complementary the 2002–2003 period for model validation. The next steps of the analysis are performed on the 1997–2001 period. The correlograms of the rate show a non-seasonal configuration with significant coefficients only in lag 1 (ACF = 0.82, PACF = 0.82, both p < 0.05) indicating that ARIMA model (1,0,0) represents the influence of each value of the rate on the following one.

The cross correlation function between monthly values of the incidence rate and temperature for twelve lags in the 1997–2001 derivation model period, only showed statistical significance in the first lag, with a coefficient of 0.25 (95%CI:0.02,0.48) for the mean minimum temperature, and a -0.16 (95%CI: -0.49, -0.02) for the mean maximum temperature. The periodograms of serial precipitation, temperature and vegetation show 12-month seasonal oscillations, therefore term (1) of the model takes the form *β *sin *(0.52 R*_*t*_*T*_*t*_*V*_*t*_*)*. Cross correlation of this term with the residual left in the rate by ARIMA(1,0,0) shows that only the lag 1 coefficient is significant, therefore the environmental influence on the malaria rate becomes *β sin(0.52R*_*t*-1_*T*_*t*-1_*V*_*t*-1_*)*. After adding the autoregressive term and the term representing seasonal influence of the environmental variables, and adjusting by linear regression, the model acquires the final form:

*I*_*t *_= *0.80I*_*t*-1 _+ *0.99*sin *(0.52R*_*t*-1 _*T*_*t*-1 _*V*_*t*-1_*)*,

where *I*_*t *_is the rate for any month *t*, *I*_*t*-1 _is the observed rate in the preceding month, *R*_*t*-1 _is the cumulative rainfall in the preceding month, *T*_*t*-1 _is the mean maximum temperature of the preceding month, and *V*_*t*-1 _is the mean vegetation density in the preceding month.

This model explains a substantial percentage of the observed variability in the malaria rate (R^2 ^_adj _= 82%, F = 165, df = 2, p < 0.0001) with a coefficient of 0.80 for the autoregressive term and 0.99 for the environmental term (Table 1). The model leaves a base rate residual in the form of white noise which is normally distributed with a mean of 0 and standard deviation of 0.98.

**Table 1:** Regression modelling results

**Model term**	**Regression Coefficient**	**SE**	**95% CI**	**p-Value**
*I*_*t*-1_	0.80	0.01	0.68 – 0.95	< 0.0001
*sin(0.52R*_*t*-1_*x T*_*t*-1_*x V*_*t*-1_*)*	0.99	0.19	0.97 – 1.00	< 0.0001

SE: Estimated regression coefficient standard error

95%CI: Confidence interval at 95% level for estimated regression coefficient

**Figure 2:**
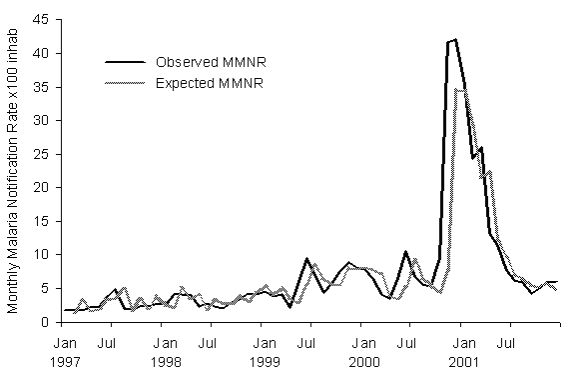
Expected and observed monthly malaria notification rates (MMNR) in the derivation period 1997–2001.

**Figure 3:**
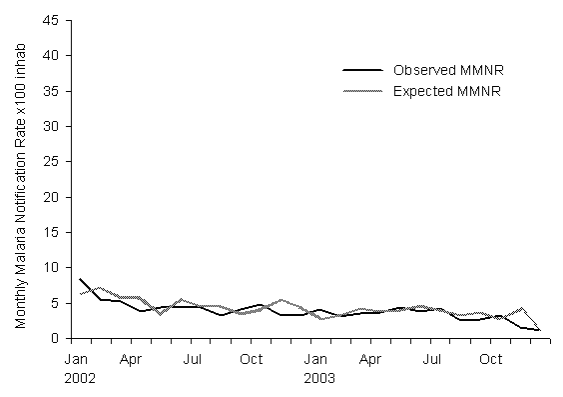
Expected and observed monthly malaria notification rates (MMNR) in the validation period 2002–2003.

The values predicted by the model and the observed values for the malaria incidence rates in 1997–2001 are shown in Table 2 and graphically in Figure 2. And the same values for the 2002–2003 period are shown on Table 3 and Figure 3. Analysis of the difference between the observed and predicted values of the malaria rate shows a periodogram with notable amplitudes in the whole frequency range; both this result and that of the runs test show typical white noise behaviour. The histogram of the differences and the Kolmogorov-Smirnov test indicate that this noise closely follows a normal probability distribution with a mean of -0.1 and standard deviation of 1.2.

**Table 2:** Expected malaria incidence by forecasting model and observed monthly malaria notification rates per 100 inhabitants of Karuzi, Burundi, in the derivation period 1997–2001.

**Month**	**Expected**	**Observed**	**Month**	**Expected**	**Observed**
Jan 1997	---	1.7	Jul	8.5	6.9
Feb	1.4	1.6	Aug	6.5	4.4
Mar	3.2	1.7	Sep	5.5	5.7
Apr	1.7	2.2	Oct	5.5	7.6
May	1.9	2.3	Nov	8	8.9
Jun	3.3	3.8	Dec	7.9	8.1
Jul	3.6	4.9	Jan 2000	8.2	7.9
Aug	4.9	2.1	Feb	7.8	6.5
Sep	1.7	1.9	Mar	7.2	4.2
Oct	3.5	2.3	Apr	4	3.5
Nov	1.9	2.4	May	3.4	6.5
Dec	3.8	2.8	Jun	5.2	10.4
Jan 1998	2.6	2.7	Jul	9.3	6.8
Feb	2.2	4.1	Aug	6.5	5.6
Mar	5	4.1	Sep	5.5	5.4
Apr	3.5	4	Oct	4.3	9.5
May	4.2	2.3	Nov	7.7	41.6
Jun	1.8	2.7	Dec	34.5	42.2
Jul	3.3	2.4	Jan 2001	34.4	35.3
Aug	2.9	2.1	Feb	29.4	24.4
Sep	2.7	2.8	Mar	21.4	25.9
Oct	3.9	3.4	Apr	22.4	13.2
Nov	3	4.3	May	12.2	11.4
Dec	4.3	4.2	Jun	9.8	7.7
Jan 1999	5.3	4.5	Jul	7.2	6.2
Feb	4.1	3.8	Aug	6.6	6
Mar	5	4.1	Sep	5.5	4.3
Apr	3.6	2.2	Oct	5.2	5
May	2.9	5.6	Nov	5.9	5.9
Jun	5.5	9.4	Dec	4.8	6

**Table 3:** Expected malaria incidence by forecasting model and observed monthly malaria notification rates per 100 inhabitants of Karuzi, Burundi, in the validation period 2002–2003.

**Month**	**Expected**	**Observed**	**Month**	**Expected**	**Observed**
Jan 2002	6.2	8.4	Jan 2003	2.7	4
Feb	7.3	5.4	Feb	3.3	3
Mar	5.8	5.2	Mar	4.3	3.4
Apr	5.8	3.8	Apr	3.9	3.6
May	3.3	4.4	May	4	4.3
Jun	5.5	4.3	Jun	4.7	3.7
Jul	4.5	4.3	Jul	4	4.1
Aug	4.5	3.1	Aug	3.3	2.4
Sep	3.5	4.1	Sep	3.7	2.6
Oct	4.1	4.7	Oct	2.7	3.1
Nov	5.5	3.2	Nov	4.2	1.5
Dec	4.4	3.2	Dec	1.3	1.1

**Figure 4:**
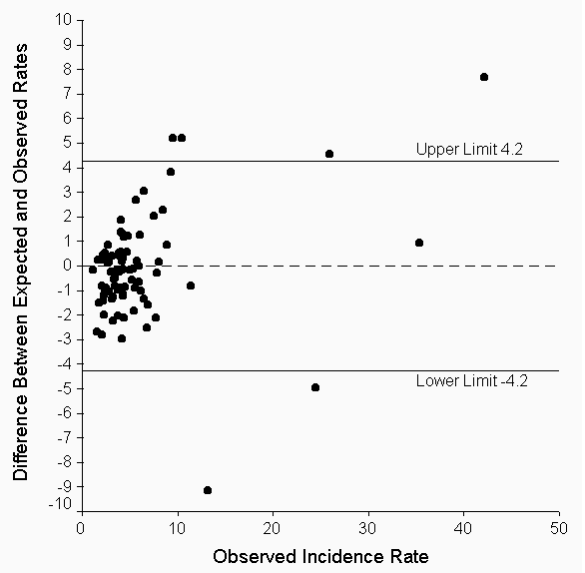
Scatterplot of difference between expected (by model) and observed malaria notification rates and observed incidence rate.

Figure 4 shows a scatterplot of the difference between the predicted and observed rates with respect to the observed rate, with the arithmetic mean and 95% confidence interval for the difference. About 4.8% (4/83) of the differences are above the 95% confidence interval of ± 4 cases per 100 inhabitants, and 2.4% (2/83) are below. The correlation between the difference and the observed rate is 0.03 (p = 0.451), and the slope of the trend of the difference with respect to the observed rate is 0.02 (p = 0.967). Taken together, these results suggest that the model can adequately forecast the monthly malaria incidence rate.

## Discussion

The model constructed in this work attempts to provide a simple tool to obtain a reliable estimate of the expected incidence of malaria one month in the future based on the observed incidence rate and a combination of climatic factors (temperature, rainfall and vegetation index) for the current month. This procedure is based on the hypothesis that the incidence rate in any particular month, provided there are no major variations on access to health services such as changes in user fees or in the criteria used for clinical and/or parasitological diagnosis, is an excellent estimator of the parasitic reservoir in a population of unstable malaria transmission, and is the most important factor affecting the rate during the following month.

Malaria Early Warning Systems (MEWS) usually monitor meteorological conditions such as rainfall and temperature; and early detection is based on routine clinical surveillance. Roll Back Malaria (RBM) has supported the development of a simple tool to monitor epidemic risks in marginal transmission areas based on anomalies in rainfall and temperature as identified from satellite observations and predictions point to at risk areas where epidemics might occur around six weeks after the detected meteorological change (the risks are displayed on maps which are updated every 10 days and can be freely accessed via the RBM or Africa Data Dissemination Service web sites). This is working well in Southern Africa but has not yet proven to be accurate in highland areas [18].

Factors related with population vulnerability are also critically important in malaria transmission. The presence of parasite resistance to the usual antimalarials and to insecticides, population movements and the presence of other underlying infections (e.g., HIV) are responsible for a large part of the variability in the incidence of malaria. Including these factors in models to predict malaria incidence is complex and not yet well understood. This has led some authors to develop models in which incidence rates are standardized with respect to non-climatic variables so that the influence of climate on fluctuations in the malaria rate can be seen more clearly [19].

The model derived in this paper implicitly assumes population vulnerability factors in the term influence of the malaria rate in the preceding month, combining it with influence of the climatic variables as factors predisposing transmission. Incidence in children less than five years of age may be a more accurate proxy for reservoir load as this age group bears the major charge of gametocytes. The results show the relation of the incidence rate with rainfall and vegetation density in the preceding month. A number of authors have found a strong correlation between the incidence rate and variations in these environmental variables during several preceding months [20], or with interannual variations in these variables [19,21]. The model derived in the present study uses the absolute value of rainfall and vegetation density, assuming that these factors have a directly proportional influence on vector density and capacity with a one month interval, which is sufficient to complete the incubation interval or minimum generation time: the complete gametocyte-to-gametocyte cycle.

A strong correlation between the incidence rate and the maximum temperature in the preceding month was found; however, no association was seen with the minimum temperature. An explanation for this finding could be that the mean minimum monthly temperature may consistently exceed the level needed for a viable sexual cycle of the parasite in the vector. Thus, the slight fluctuations observed at temperatures higher than that level do not have a significant influence on the extrinsic incubation period, in contrast to what occurs with the maximum temperature. The situation may be different in higher altitude areas, where variations in the minimum temperature under 10°C could block the sexual cycle of *P. falciparum*, and significantly affect incidence levels and cycles, population immunity and the age-distribution of cases.

Extrapolation of the results obtained in this work can neither be affirmed nor ruled out to other areas of equal altitude for a particular latitude (where rainfall is an important factor) or even for similar latitudes with different epidemiologic factors, for example, the parasite species and the characteristics of the local vector species. Other authors who have developed transmission models have noted the importance of maximum ambient temperature in malaria transmission in certain areas [22]. The model created by Loevinsohn [20] for an area of unstable malaria transmission in Rwanda included minimum temperature in the preceding one and two months, and rainfall in the preceding two and three months, and was found to explain a considerable part of the variability in malaria incidence. This author did not take into account the influence of the malaria incidence rate in the preceding month. In the method used by Abeku *et al *[23], the variable malaria incidence was log transformed to avoid the potential problems related with techniques that assume normally distributed data. However, it seems that this procedure reduces the sensitivity and the transparency of the resulting model.

This study is subject to various limitations. The first is the assumption that data collection methods did not change during the study period; in a 7-year period there could well have been changes in the way cases were reported and registered. The second limitation arises from the definition of a case as notification of a patient consultation for malaria without microbiological confirmation in most cases, which affects the representativeness of the system. In this regard, what is important is not to represent the volume of what is really occurring, but rather the upward or downward movements that can be observed with the historic stability of this form of reporting.

Another limitation is that rainfall and temperature data were gathered in the only station with measuring instruments located in the same place. It is true that the geographical diversity of Burundi can mean a limitation due to fluctuation of these variables in small distances, but it was accepted to gather these data for the following reasons: a) the record of these variables from 1997 to 2003 has remained uniform, with the same measurement instruments, the same calibration and the same precision; b) impossibility to have access to instrumental records in different parts of the Karuzi province during the research period; and c) other authors have used environmental variables with similar or greater space resolution than the one used for this research in environments with similar geographical diversity to study its influence in the epidemiology of transmissible diseases and/or creation of incidence prediction models bearing in mind such variables [19,24]. The fourth limitation is that in the model created the minimum surveillance period for the detection of substantial variations in the incidence is one month. As with most epidemiological surveillance systems in rural areas of Africa, it is very difficult to obtain weekly reporting of malaria cases, which would permit monitoring of shorter time intervals and an earlier system of alert; this is what health facilities in these areas should perform for an opportune surveillance. Teklehaimanot et al [25] have proposed and evaluated these types of models using as entry data weekly reports of both malaria cases and the percentages of positive parasitemia in areas of Ethiopia, but their models do not take into account the influence of climatic factors, which are difficult to obtain with this periodicity.

Despite these limitations, the model derived in this paper may give a more accurate prediction of malaria epidemics by taking into account a readily available proxy (previous month incidence) for malaria reservoir, combined with key environmental factors. However, that information would narrow the prediction time of six weeks of the environmental-based MEWS and would require improved communication and reaction strategies.

## Conclusion

In summary, in this paper it has been developed a model to predict the expected incidence of malaria in a highland area of Africa one month in the future based on the observed incidence rate and a combination of climatic factors (temperature, rainfall and vegetation index) for the current month. Although the model is reasonably reliable, especially with regard to the magnitude of the prediction, it requires active field evaluation to test its behaviour in real life situations. The model is open to modification to try to achieve adequate and timely forecasting of malaria epidemics, with the ultimate aim of reducing the suffering caused by this disease in inhabitants of the African highlands.

## Authors' contributions

AGE conceived and coordinated the study, participated in the design, was involved in the interpretation of results, and helped to draft the manuscript. AO participated in the design of the study and assisted in drafting the manuscript. MVH made data available in collaboration with the Ministry of Health of Burundi, was involved in the initial design of the study and critical review of the manuscript. AAJ participate in the conception, performed the statistical analysis, was involved in the interpretation of results and participated in drafting the manuscript. All authors have read and approved the submitted version of the manuscript.

## References

[B1] WHO/UNICEF (2005). The world malaria report 2005.

[B2] WHO (2004). The World Health Report 2004: Changing History.

[B3] Nájera JA, Kouznetzsov RL, Delacollette C (1998). Malaria epidemics. Detection and control, forecasting and prevention. WHO/MAL/981084.

[B4] Worrall E, Rietveld A, Delacollette C (2004). The burden of malaria epidemics and cost – effectiveness of interventions in epidemic situations in Africa. Am J Trop Med Hyg.

[B5] WHO (2004). Malaria Epidemics: Forecasting, Prevention, Early Detection and Control. From policy to practice. Report of an Informal consultation, Leysin, Switzerland 8–10 December 2003.

[B6] WHO (2001). Malaria Early Warning Systems: concepts, indicators and partners: A framework for field research in Africa.

[B7] WHO (2002). Final report on the 3rd meeting of the RBM Technical Resource Network on Epidemic Prevention and Control.

[B8] Kilian AHD, Langi P, Talisuna A, Kabagambe G (1999). Rainfall pattern, El Niño and malaria in Uganda. Trans R Soc Trop Med Hygiene.

[B9] Hay S, Were E, Renshaw M, Noor AM, Ochola S, Olusanmi I (2003). Forecasting, warning, and detection of malaria epidemics: a case study. Lancet.

[B10] Abeku TA, van Oortmarssen GJ, Borsboom G, de Vlas SJ, Habbema JD (2003). Spatial and temporal variations of malaria epidemic risk in Ethiopia: factors involved and implications. Acta Trop.

[B11] Thomson MC, Connor SJ, Milligan PJW, Flasse S (1997). Mapping malaria risk in Africa – what can satellite contribute?. Parasitol Today.

[B12] Hay SI, Snow RW, Rogers DJ (1998). From predicting mosquito habitat to malaria seasons using remotely sensed data: practice, problems and perspectives. Parasitol Today.

[B13] Guthmann JP, Bonnet M, Ahoua L, Dantoine F, Balkan S, van Herp M, Tamrat A, Legros D, Brown V, Checchi F (2007). Deaths rates from malaria epidemics, Burundi and Ethiopia. Emerg Infect Dis.

[B14] Legros D, Dantoine F (2001). Epidémie de paludisme du Burundi Septembre 2000 – Mai 2001.

[B15] United Nations Population Division World Population Prospects: The 2006 Revision. http://esa.un.org/unpp.

[B16] Africa Data Dissemination Service Dekadal Normalized Difference Vegetation Index. http://earlywarning.usgs.gov/adds/.

[B17] MacDonald G (1957). The epidemiology and control of malaria.

[B18] Grover-Kopec E, Kawano M, Klaver RW, Blumenthal B, Ceccato P, Connor SJ (2005). An online operational rainfall-monitoring resource for epidemic malaria early warning systems in Africa. Malar J.

[B19] Thomson MC, Mason SJ, Phindela T, Connor SJ (2005). Use of rainfall and sea surface temperature monitoring for malaria early warning in Botswana. Am J Trop Med Hyg.

[B20] Loevinsohn ME (1994). Climatic warming and increased malaria incidence in Rwanda. Lancet.

[B21] Zhou G, Minakawa N, Githeko AK, Yan G (2004). Association between climate variability and malaria epidemics in the East African highlands. Proc Natl Acad Sci USA.

[B22] Hoshen MB, Morse AP (2004). A weather – driven model of malaria transmission. Malar J.

[B23] Abeku TA, De Vlas SJ, Borsboom GJ, Tadege A, Gebreyesus Y, Gebreyohannes H, Alamirew D, Seifu A, Nagelkerke NJ, Habbema JD (2004). Effects of meteorological factors on epidemic malaria in Ethiopia: a statistical modelling approach based on theoretical reasoning. Parasitology.

[B24] Craig MH, Kleinschmidt I, Nawn JB, Le Sueur D, Sharp BL (2004). Exploring 30 years of malaria data in KwaZulu-Natal, South Africa: Part I. The impact of climatic factors. Trop Med Int Health.

[B25] Teklehaimanot HD, Schwartz J, Teklehaimanot A, Lipsitch M (2004). Alert threshold algorithms and malaria epidemic detection. Emerg Infect Dis.

